# Trained Quantity Abilities in Horses (*Equus caballus*): A Preliminary Investigation

**DOI:** 10.3390/bs4030213

**Published:** 2014-07-25

**Authors:** Maria Elena Miletto Petrazzini

**Affiliations:** Department of General Psychology, University of Padova, Via Venezia 8, 35131 Padova, Italy; E-Mail: mariaelena.milettopetrazzini@unipd.it; Tel.: +39-049-827-7424; Fax: +39-049-827-6600

**Keywords:** *Equus caballus*, quantity discrimination, continuous quantities

## Abstract

Once believed to be a human prerogative, the capacity to discriminate between quantities now has also been reported in several vertebrates. To date, only two studies investigated numerical abilities in horses (*Equus caballus*) but reported contrasting data. To assess whether horses can be trained to discriminate between quantities, I have set up a new experimental protocol using operant conditioning. One adult female was trained to discriminate between 1 and 4 (Test 1) in three different conditions: non-controlled continuous variables (numerical and continuous quantities that co-vary with number are simultaneously available), 50% controlled continuous variables (intermediate condition), and 100% controlled continuous variables (only numerical information available). The subject learned the discrimination in all conditions, showing the capacity to process numerical information. When presented with a higher numerical ratio (2 *vs.* 4, Test 2), the subject still discriminated between the quantities but its performance was statistically significant only in the non-controlled condition, suggesting that the subject used multiple cues in presence of a more difficult discrimination. On the whole, the results here reported encourage the use of this experimental protocol as a valid tool to investigate the capacity to process numerical and continuous quantities in horses in future research.

## 1. Introduction

Quantitative ability in non-human animals represents one of the topics most investigated in comparative psychology during the last decade. Today we know that there are several natural contexts in which the capacity to discriminate between quantities can enhance survival. Such abilities are useful to establish in social interactions when attacking other conspecifics; chimpanzees and hyenas, for instance, are more likely to attack other groups of conspecifics when they perceive themselves as being part of a larger group [1,2]. Quantity abilities are useful also in anti-predator defense, as many social species take advantage by joining the largest available group of conspecifics when chased by predators (the so-called “safety-in-numbers” effect [3]). Other potential advantages are related to food choice (e.g., selection of the larger quantity of food items [4,5]) and mate choice (e.g., selection of the larger group of potential mates [6]). 

Laboratory studies found that vertebrates as diverse as mammals (orangutans [7], gorillas [8], bears [9], striped field mice [10]), birds (parrots [11], chicks [12]), fish (guppies [13], angelfish [14]) and also some invertebrates (e.g., bees [15,16] and ants [17]) can discriminate between quantities in several contexts. However, numerical information usually co-varies with other continuous attributes of the stimuli, such as density of the objects, cumulative surface area or the overall space occupied by the sets. These non-numerical variables, commonly defined as “continuous quantities” [18,19], are taken into account in most of these studies in order to assess which is the exact cognitive mechanism utilized by animals in their quantity judgments. For instance, it was demonstrated that cats are able to learn to discriminate between two and three bi-dimensional figures to get a food reward. However, as soon as the stimuli were controlled for cumulative surface area (by enlarging the size of items in the smaller group and reducing those included in the larger group, so that the sum of areas was identical in the two groups), cats’ performance dropped to chance level. This suggests that cats can discriminate between quantities but primarily base their choices on continuous quantities instead of numbers [20]. Other studies that used real objects, instead of bi-dimensional stimuli, faced similar methodological issues. Kilian and colleagues [21] trained a dolphin to discriminate between two and five three-dimensional objects. Once the subject learned the discrimination, new spatial configurations were introduced to assess whether the subject based its choice on elements’ pattern rather than on number. Results showed a decrease in the subject’s performance, thus providing evidence of the salience of non-numerical cues on dolphin’s quantity abilities. Conversely, rhesus monkeys proved able to discriminate between two sets of lemons matched for total contour length and volume but differing in numerosity, suggesting the existence of spontaneous numerical abilities in this species [22].

To date, there are broad phylogenetic assessments of various quantitative and numerical abilities in animals. Most of the studies focused on traditional lab species, such as macaques [23,24,25] and pigeons [26,27], while little research has been reported with species that are rare, endangered or that cannot be easily maintained in laboratory. For instance, elephants’ numerical abilities have been investigated in three studies [5,28,29], only one study was reported on raccoons [30] as well as a single study which investigated sea lions [31]. Multiple investigations in the same species is, of course, fundamental to broaden our comprehension of numerical abilities of those animals, but—for a larger scale view of vertebrates’ numerical abilities—we would benefit from encompassing species that have been seldom investigated until now, especially if researchers aim to assess similarities and differences of quantity abilities among vertebrates.

With respect to this topic, horses represent one of the species whose quantity abilities are still largely unknown even though also horses might take advantage of such cognitive skills. For instance, joining a larger group of conspecifics seems to decrease the individual’s level of fly harassment in feral horses [32]. Also, quantity information might be useful to guide foraging decisions (e.g., finding the larger quantity of food items), or to select the most advantageous social group in terms of sex-ratio, as commonly reported in other vertebrates [4,5,6]. Alternatively, the possibility exists that vertebrates share a core numerical system inherited by a common ancestor (e.g., [33]). For instance, a recent study found that even blind cavefish that lived in the darkness of Somalia caves in the total absence of predators for approximately two million years display numerical abilities partially similar to those described in other fish species [34]. Similarly, horses might exhibit some rudimentary numerical abilities irrespective to the ecological pressures received across evolution.

To date, with the exception of the well-known example of “Clever Hans” [35], a case that was criticized on several grounds in the past for its lack of control, only two studies have been reported in the literature. Uller and Lewis [36] provided the first evidence of quantity discrimination abilities in horses. The authors used a spontaneous choice task in which horses could choose between two quantities of apples sequentially introduced into two opaque containers. The whole sets were not visible at the time of choice, thus preventing the possibility that horses could use any currently available visual cue of full continuous amounts of food to make their choices. The horses selected the larger number of apples in 1 *vs.* 2 and 2 *vs.* 3 but not in 4 *vs.* 6. Interestingly, when the subjects were presented with a single large apple and two small apples, they still chose the larger number of items, proving the ability to base their choice on number rather than on volume. However, recently Henselek, Fischer, and Schloegl [37] did not find similar capacities in horses. In this study, subjects were given the choice between two sets of items differing in numerosity and presented simultaneously in three experimental conditions. In two conditions, food was used as a stimulus choice because it is known that several animals spontaneously select the larger amount of food when presented with two alternatives (for a review see [38]). In particular, in the “food” condition, apple slices were used as stimuli and as reward, and in the “food replaced” condition, the choice was between two sets of apple slices, but the subjects received other apple slices as reward. In the third condition (“wood condition”), no food but rather wooden blocks were presented as stimuli, and the horses were rewarded with the corresponding number of apple slices. Overall, the horses proved unable to discriminate the numerical contrasts presented in all experimental conditions, both when they could make a spontaneous choice between edible food and when they were required to associate a certain number of objects with the corresponding number of food items. 

In sum, while one study suggested numerical discrimination in horses, another study did not report a similar outcome. Part of the inconsistencies reported in the two studies might be ascribed to the different methodology adopted, such as different paradigms (e.g., sequential *vs.* simultaneous presentation), stimuli (e.g., entire apples *vs.* apple slices) and number of trials [39]. There is indeed evidence that different methods of measuring quantitative abilities can lead to different results in the same species. Goldbelly topminnows could discriminate up to 2 *vs.* 3 companions with one experimental procedure while they were unable to solve the same task when a different procedure was used [40]. 

With respect to this topic, two main methodological approaches have been used to study quantity abilities in non-human animals: spontaneous choice tests and training procedures [38]. The former approach consists of presenting two groups of biologically-relevant stimuli (e.g., food items, potential preys *etc.*) differing in quantity, with the assumption that if subjects are able to discriminate between the two quantities, they are expected to select the larger/smaller quantity. In the latter approach, subjects undergo extensive training in which some neutral stimuli (e.g., dots) are associated with a reward and the capacity to learn a numerical rule is taken as evidence of numerical abilities. This approach often requires the subjects to learn a numerical rule (e.g., selecting the larger number of dots in order to obtain a food reward) that cannot be compared to most of the problems faced in nature. As a consequence, studies using training procedures commonly lack ecological relevance. Nonetheless, training procedures present unquestionable advantages compared to spontaneous choice tests. In spontaneous choice tests, motivation plays a key-role and null results do not necessarily imply a lack of discrimination. For instance, subjects may not select the larger group of apples in 5 *vs.* 6 discrimination simply because both groups are large enough to satisfy them. Also, controls for continuous quantities are always difficult with biologically-relevant stimuli used in spontaneous choice tests (olfactory cues *in primis*). These issues can be tackled by using training procedures in which subjects are constantly motivated by rewarding the correct choice, regardless of the numerosity of the items and in the total absence of olfactory cues.

To date, no study used training procedures based on operant conditioning to investigate numerical competence in horses. The present study aimed to investigate quantity discrimination in horses by using an operant conditioning procedure. For this purpose, I have used an experimental design frequently reported in comparative psychology to test quantity abilities of species that cannot be easily tested and/or kept in laboratories (e.g., orangutan [7], bear [9], elephant [5], dolphin [21]). In these studies, a single or a reduced number of individuals is tested, with the assumption that if at least one individual can be successfully trained, the cognitive system of the species investigated is fully equipped to solve the task [41]. As a consequence, I have trained a single horse to discriminate between 1 *vs.* 4 (Test 1) and 2 *vs.* 4 (Test 2). In both tests, the subject could reach a food reward only by selecting the larger quantity. To assess the role of continuous quantities in this task, three different conditions were presented: non-controlled stimuli (number + continuous quantities available), 50% controlled stimuli (intermediate condition), and 100% controlled stimuli (pure numerical discrimination).

## 2. Method

### 2.1. Subject

An adult female (age: 10 years) of domestic horse (*Equus caballus*), named Shanty, was tested as subject. She was housed at a private riding stable located in Ferrara di Montebaldo (Verona, Italy), where experiments were carried out between June and September 2012. Shanty was not deprived of food in any phase of the project, nor forced to participate in the experiment when unwilling to do so. The owner gave her written consent before starting the experiment.

### 2.2. Apparatus and Stimuli

The experimental apparatus consisted of two wooden panels (175 × 80 × 31 cm each) separated by a green plastic divider (poliplack © 149 × 56 cm). In both panels, there was a window (53 × 40 cm) centrally located and placed to 55 cm from the ground, in correspondence of which two panels of transparent plastic frames hosted the stimulus sheets. 

**Figure 1:**
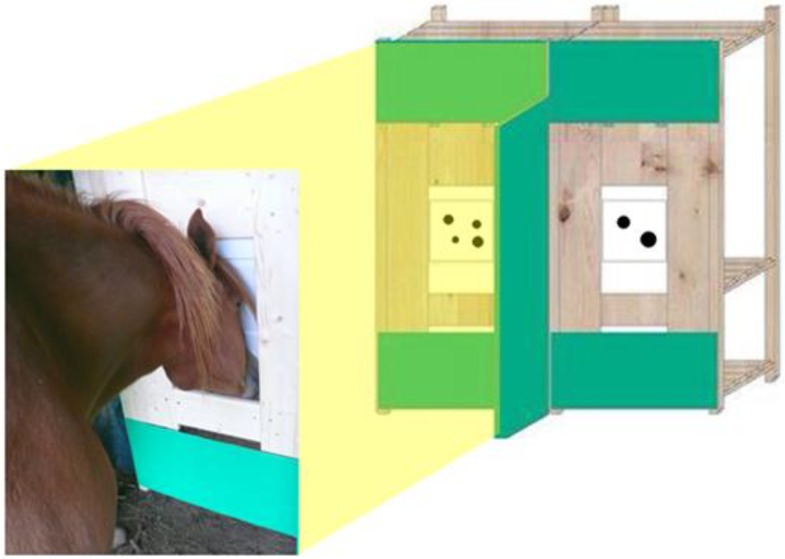
The subject was required to touch with the snout one of two stimulus panels in order to obtain a food reward. Food was available behind each panel in order to control for olfactory cues, but only the panel associated with the larger group could be bent to reach the food.

The upper part of the panels was fixed to the wood structure so that the horse could open them by pushing them with the snout. Two bowls containing food (pieces of carrots or apple slices) were placed behind each window. The panel associated with the reinforced quantity could be easily opened by the horse; on the contrary, the panel associated with the non-reinforced quantity could not be open as it was blocked by a wooden beam from behind. In this way, olfactory cues were available on both sides of the apparatus and could not bias the subject (Figure 1). A video camera placed behind the subject was used to record the experiment.

Stimuli used during the familiarization phase consisted of two identical black rectangles (15.7 × 6.7 cm) on a white background (42 × 29.7 cm): on one side, a horizontal rectangle was presented; on the other side, a vertical rectangle was presented.

In the training phase, the stimuli were composed of groups of black dots (ranging from 7.3 × 2.2 cm) on a white background (42 × 29.7 cm). I tested the subject until it was no longer available. For this reason two different numerical ratios were presented: 1 *vs.* 4 (Test 1) and 2 *vs.* 4 (Test 2), with 24 stimulus pairs for each test. Spatial configurations of dots were changed both within and between sets in both numerical contrasts. As a consequence, all stimulus pairs differed for their pattern configuration to avoid the possibility that the horse could have learned the discrimination on the basis of the element pattern.

To assess the role of numerical and continuous information in quantity discrimination, three different conditions were set up for each test: non-controlled, 50% controlled and 100% controlled. In the first condition, number and continuous quantities were simultaneously available. In short, the ratio between the cumulative surface area in a 2 *vs.* 4 discrimination was equal to 2/4 (congruent with the numerical ratio). In the 100% controlled condition, the cumulative surface area was equated between the two groups by enlarging the stimulus size of the dots included in the smaller group and reducing the size of the dots included in the larger group. The 50% condition represented an intermediate circumstance in which cumulative surface area was only partially controlled. For instance, in a 2 *vs.* 4 discrimination, the ratio of cumulative surface areas between the smaller and larger group was equal to 0.75. Dot size was also varied across sets to reduce the possibility that Shanty could have used this non-numerical cue to discriminate between quantities. In particular, the largest dot was presented in the larger group in half of the trials and in the smaller group in the other half of the trials. 

In addition, in 2 *vs.* 4, half of the stimuli were controlled for the overall space encompassed by the most lateral items of the arrays and half of the stimuli were controlled for inter-item distance. In 1 *vs.* 4, however, cumulative surface area of the smaller group corresponded to the overall space occupied by the group itself and no inter-item distance could be calculated in this group (in short, the three continuous quantities collapsed in a single variable). This type of control for continuous quantities has been previously used in other studies (e.g., [42,43]).

Cumulative surface area, inter-item distance and overall space occupied by the two arrays were controlled by using TpsDig software.

### 2.3. Procedure

The procedure consisted of two phases: (a) familiarization and (b) training. The former was set up to permit the subject to become familiar with the experimental apparatus and the motor response required to reach the food reward. A non-numerical task was presented in this phase to avoid any interference with the subsequent numerical learning. In the latter phase, the subject was trained to discriminate between two different numerical contrasts. Both phases were conducted in a riding area familiar to the horse outside of the stable where Shanty was housed.

#### 2.3.1. Familiarization

This phase was set up to familiarize the horse with the apparatus and the procedure used. Two experimenters were involved: Experimenter 1 (E1), who kept the horse at a distance of 1.5 m from the apparatus in a central position, and Experimenter 2 (E2), who was hidden behind the apparatus and therefore could not cue the subject when she changed the stimuli.

The experimenter handling the horse could change between sessions; however, the person was always instructed to avoid giving any cue to the horse during the trials. 

In the first trials, both panels were kept slightly open and no stimulus was presented to accustom the horse to put its head in the two windows and be able to access the reward (pieces of apple or carrots) in both bowls. At the beginning of each trial, the experimenter hidden behind the apparatus attracted the attention of the horse by calling its name, and then the handler left the subject free to approach the apparatus and make its choice. After each trial, E1 led the horse in a semicircle to the left or the right in order to return to the starting point.

Once the subject had become familiar with the apparatus by placing the snout twice in both windows, both panels were completely closed and the procedure was repeated until the horse learned to open them at least twice on each side. Subsequently, the horse was trained to choose only one of two stimuli (vertical rectangle *vs.* horizontal rectangle) to habituate the subject to the fact that one stimulus gave access to food and the other one was not associated with food. At the beginning of each trial, E2 inserted simultaneously the pair of stimuli in the two panels and called the horse by name to be sure it was paying attention to the stimuli; then E1 released the subject. To avoid the development of side bias, prior to the experiment, the position of the reinforced stimulus was pseudo-randomly determined to ensure that it was never shown more than twice in a row on the same side and to counterbalance the left-right presentations over trials.

Each session consisted of 14 consecutive trials, and Shanty was subjected to one session per day. When the horse chose the correct stimulus (vertical rectangle) by pushing the corresponding panel, it was allowed to eat the food reward, and a secondary reinforcement was given by E2 saying “Well done!”. If Shanty chose the incorrect stimulus (horizontal rectangle), she could not insert her snout as the window was locked and the hidden experimenter said “No!” as a secondary punishment; the horse was then led to the starting point.

To avoid Shanty focusing on horizontal *vs.* vertical displacement of the objects presented in the next training phase (a useless cue in the quantity task), a low threshold was set up for moving forward to the training phase, consisting in 55% (eight out of 14) correct choices over two consecutive days.

#### 2.3.2. Training

The subject was trained to choose the larger between two sets of two-dimensional stimuli differing in number. The procedure was the same as previously used for the discrimination of rectangles, but each session consisted of 12 trials. Two different tests were planned. 

Test 1: Shanty was initially trained to discriminate between 1 and 4 dots. One third of the stimuli presented in each session was controlled for the cumulative surface area (100%), another third was not controlled and the remaining third was controlled to 50%. The three conditions were randomly presented during each session to avoid the horse learning to use alternative strategies to solve the task (for instance, if the three conditions would have run in separate sessions, the horse might have noticed that in the 100% controlled condition the reinforced group was composed, on average, by the smallest objects in the two arrays, and she might have used this cue instead of number). The learning criterion was set at 75% (nine out of 12) correct trials over two consecutive days (corresponding to a statistically significant preference with the chi-square test calculated on the two days), as commonly done in training studies of mammals (see [38]).

Test 2: Once the criterion was reached, the horse was trained to discriminate a novel contrast with reduced numerical distance: 2 *vs.* 4. The same procedure adopted in Test 1 was used. The learning criterion was again set at 75% (nine out of 12) correct trials over two consecutive days.

Statistical analyses were performed using the statistical software SPSS 19.0.

## 3. Results

### 3.1. Test 1: 1 vs. 4 Discrimination

**Figure 2:**
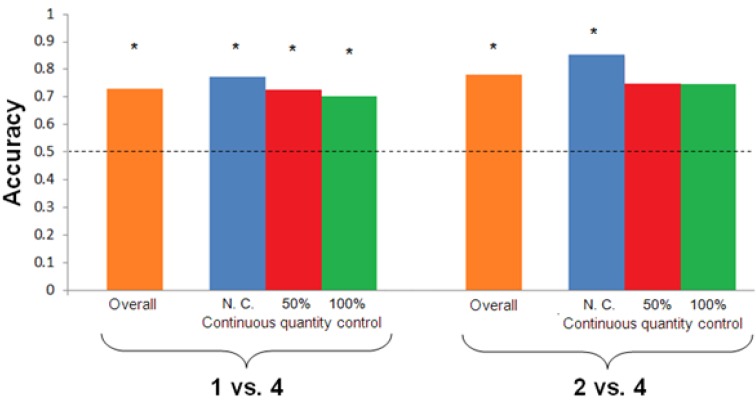
Accuracy (proportion of correct choices) is plotted against the type of stimuli (non-controlled, 50% controlled, 100% controlled stimuli) separately for each Test. Asterisks (*) denote a significant departure from chance level. Horizontal dashed line indicates chance level.

Shanty reached the learning criterion after 132 trials. The frequency of correct choices was statistically significant (chi-square, χ_(1)_ = 29.121, *p* < 0.001, Figure 2). In particular, Shanty proved able to discriminate the two quantities in all of the conditions (non-controlled stimuli: χ_(1)_ = 13.091, *p* < 0.001; 50% controlled stimuli χ_(1)_ = 9.091, *p* = 0.003; 100% controlled stimuli χ_(1)_ = 7.364, *p* = 0.007). No left-right bias was observed (proportion of left choices, 0.59; proportion of right choices, 0.41, χ_(1)_ = 2.979, *p* = 0.084).

### 3.2. Test 2: 2 vs. 4 Discrimination

Shanty reached the learning criterion after 24 trials. The frequency of correct choices was statistically significant (chi-square, χ_(1)_ = 8.167, *p* < 0.004, Figure 2). However, a significant discrimination was observed in the non-controlled condition (χ_(1)_ = 4.500, *p* = 0.034) but not in the other two conditions (50% controlled stimuli χ_(1)_= 2.000, *p* = 0.157; 100% controlled stimuli χ_(1)_ = 2.000, *p* = 0.157). Again, no left-right bias was observed (proportion of left choices: 0.53; proportion of right choices: 0.47, χ_(1)_ = 0.053, *p* = 0.819).

## 4. Discussion

The present study aimed to test whether horses can be trained to discriminate between quantities. For this purpose, I adapted the two-choice discrimination task commonly used in mammals [9,19,21], presenting two numerical contrasts and using bi-dimensional stimuli that permit a fine-grained manipulation of continuous quantities. 

Test 1 showed that Shanty was able to discriminate 1 *vs.* 4 in all conditions. In particular, she successfully discriminated the two quantities also when the use of continuous quantities was experimentally limited, thus suggesting the existence of trained numerical discrimination in this species. It must be noted, however, that a 0.25 ratio represents a potentially easy numerical ratio discriminated also by basal vertebrates and invertebrates (e.g., angelfish, guppies [14,43] and bees [16]). Therefore, this result, although new in the horse literature, may not be unexpected.

Test 2 aimed to assess whether the subject could also discriminate a higher ratio, 2 *vs.* 4 (0.50). Shanty proved able to discriminate also between these two quantities after only 24 trials. The fact the she needed a reduced number of trials to reach the learning criterion compared to Test 1, where a potentially easier numerical ratio was presented (18% of overall trials compared to Test 1), is indicative of the fact that she generalized the quantity/numerical rule learned in the previous test. This result should not be neglected when searching for the most proper procedure to study trained numerical/quantity abilities in horses: with the experimental protocol here adopted, very easy ratios can be presented at the beginning with the aim to introduce the numerical rule, similarly to what was reported in other mammals [7,9,44].

The results of Test 2, however, showed that Shanty can significantly discriminate between the two quantities only when number and continuous quantities are simultaneously available. Even though an extensive investigation of the relation between numerical and continuous information was not a primary goal of this preliminary study, the comparison of the results of the two tests provides indirect clues for the numerical cognition literature. Previous studies showed that human [45,46] and non-human animals [20,47] sometimes make use of multiple cues to discriminate between quantities. A recent paper showed that mosquitofish are better able to discriminate between 2 and 3 objects if number and continuous quantities are simultaneously available. Discrimination based on number only and continuous quantities only were equally difficult, suggesting that number is not more cognitively demanding than continuous quantities [18,48]. The performance exhibited by Shanty in the two tests suggests that she can process numerical information (Test 1), but she is facilitated by the simultaneous presence of numerical and continuous information when a harder ratio is presented (Test 2). Thus, it seems she can process both types of information at least for easy discriminations. It is possible that, once the task increases in difficulty, multiple cues (e.g., number + area) are fed into the same cognitive system to better achieve the task. After all, as stated by Gebuis and Reinvoet [45], the relation between number and continuous quantities is unlikely to be violated in nature, and there is no reason to believe that horses, as well as other animals, would equally perform in the presence of “unnatural” stimuli (such as 100% controlled stimuli) compared to more “natural” stimuli (where number and continuous quantities positively co-vary), especially with difficult discriminations. However, as Shanty reached the learning criterion in Test 2 after a smaller number of trials, the possibility exists that the different performance reported in Test 1 and Test 2 with stimuli controlled for continuous quantities may be partially ascribed to different number of trials reported in the two tests.

I am aware that these are speculations that only future studies could address. However, in recent years, an increasing number of studies testing a single animal was able to open a wide debate in comparative psychology (*i.e.*, numerical abilities: one chimpanzee [49], one parrot [11]; prospective memory: one chimpanzee [50]; rhythm perception: one parrot [51]). In this sense, results of single-case studies should not be underestimated in animal cognition, as well as the study of single cases is widely recognized as a fundamental part of the assessment process in other research fields, such as cognitive psychology and neuropsychology (*i.e.*, [52,53]).

In conclusion, although this study has several limitations (sample size and reduced number of ratios presented *in primis*), it shows that horses can be trained using operant conditioning to discriminate between quantities/numerosities using bi-dimensional stimuli. The new experimental protocol is similar to that adopted in other mammals, thus permitting a cross-species comparison between horses and other mammals, and seems to have great promise as a tool to address other unsolved issues in numerical cognition. For instance, some authors suggest the existence of two different numerical systems both in human and in non-human animals: a precise system for small (<4) numbers, called “subitizing”, and an approximate system for larger (>4) numbers, called “approximate number system” (reviewed in [33]). The conclusions of this study—as well as the conclusions made by Uller and Lewis (1 *vs.* 2 and 2 *vs.* 3, [36])—are confined in the so-called subitizing range (1–4). In order to develop a broader knowledge of quantity abilities in horses, future studies are now required to extend their investigation also beyond the 4-unit limit. Other questions are unanswered. For instance, a large debate surrounds the role of domestication in cognitive abilities [54,55]: do domesticated horses have similar performance in this quantity task compared to their “wild cousins”? Also, what are the developmental trajectories of numerical abilities in horses? Are there individual differences in horses’ quantity abilities? I am confident research will address these questions in the near future. 

## References

[1] Wilson M.L., Britton N.F., Franks N.R. (2002). Chimpanzees and the mathematics of battle. Proc. R. Soc. B.

[2] Benson-Amram S., Heinen V.K., Dryer S.L., Holekamp K.E. (2011). Numerical assessment and individual call discrimination by wild spotted hyenas, *Crocuta crocuta*. Anim. Behav..

[3] Hager M.C., Helfman G.S. (1991). Safety in numbers: Shoal size choice by minnows under predatory threat. Behav. Ecol. Sociobiol..

[4] Beran M.J., Evans T.A., Harris E.H. (2008). Perception of food amounts by chimpanzees based on the number, size, contour length and visibility of items. Anim. Behav..

[5] Irie-Sugimoto N., Kobayashi T., Sato T., Hasegawa T. (2009). Relative quantity judgment by Asian elephants (*Elephas maximus*). Anim. Cogn..

[6] Shifferman E.M. (2012). It’s all in your head: The role of quantity estimation in sperm competition. Proc. R. Soc. B.

[7] Vonk J. (2014). Quantity matching by an orangutan (*Pongo abelii*). Anim. Cogn..

[8] Vonk J., Torgerson-White L., McGuire M., Thueme M., Thomas J., Beran M.J. (2014). Quantity estimation and comparison in western lowland gorillas (*Gorilla gorilla gorilla*). Anim. Cogn..

[9] Vonk J., Beran M.J. (2012). Bears ‘count’ too: Quantity estimation and comparison in black bears, *Ursus americanus*. Anim. Behav..

[10] Panteleeva S., Reznikova Z., Vygonyailova O. (2013). Quantity judgments in the context of risk/reward decision making in striped field mice: first “count”, then hunt. Front. Psychol..

[11] Pepperberg I.M., Carey S. (2012). Grey parrot number acquisition: The inference of cardinal value from ordinal position on the numeral list. Cognition.

[12] Rugani R., Fontanari L., Simoni E., Regolin L., Vallortigara G. (2009). Arithmetic in newborn chicks. Proc. R. Soc. B.

[13] Agrillo C., Miletto Petrazzini M.E., Bisazza A. (2014). Numerical acuity of fish is improved in the presence of moving targets, but only in the subitizing range. Anim. Cogn..

[14] Gómez-Laplaza L.M., Gerlai R. (2013). The role of body surface area in quantity discrimination in angelfish (*Pterophyllum scalare*). PloS One.

[15] Pahl M., Si A., Zhang S. (2013). Numerical cognition in bees and other insects. Front. Psychol..

[16] Gross H.J., Pahl M., Si A., Zhu H., Tautz J., Zhang S. (2009). Number based visual generalisation in the honeybee. PLoS One.

[17] Reznikova Z., Ryabko B. (2011). Numerical competence in animals, with an insight from ants. Behaviour.

[18] Agrillo C., Piffer L., Bisazza A. (2011). Number *versus* continuous quantity in numerosity judgments by fish. Cognition.

[19] Cantlon J.F., Brannon E.M. (2007). How much does number matter to a monkey (*Macaca mulatta*)?. J. Exp. Psychol.: Anim. Behav. Proc..

[20] Pisa P.E., Agrillo C. (2009). Quantity discrimination in felines: A preliminary investigation of the domestic cat (*Felis silvestris catus*). J. Ethol..

[21] Kilian A., Yaman S., von Fersen L., Güntürkün O. (2003). A bottlenose dolphin (*Tursiops truncatus*) discriminates visual stimuli differing in numerosity. Learn. Behav..

[22] Flombaum J.I., Junge J.A., Hauser M.D. (2005). Rhesus monkeys (*Macaca mulatta*) spontaneously compute addition operations over large numbers. Cognition.

[23] Cantlon J., Brannon E.M. (2006). Shared system for ordering small and large numbers in monkeys and humans. Psychol. Sci..

[24] Livingstone M.S., Pettine W.W., Srihasam K., Moore B., Morocz I.A., Lee D. (2014). Symbol addition by monkeys provides evidence for normalized quantity coding. Proc. Natl. Acad. Sci. USA.

[25] Jordan K.E., Brannon E.M. (2006). Weber’s Law influences numerical representations in rhesus macaques (*Macaca mulatta*). Anim. Cogn..

[26] Emmerton J., Renner J.C. (2006). Scalar effects in the visual discrimination of numerosity by pigeons. Learn. Behav..

[27] Scarf D., Hayne H., Colombo M. (2011). Pigeons on par with primates in numerical competence. Science.

[28] Irie N., Hasegawa T. (2012). Summation by Asian elephants (*Elephas maximus*). Behav. Sci..

[29] Perdue B.M., Talbot C.F., Stone A.M., Beran M.J. (2012). Putting the elephant back in the herd: Elephant relative quantity judgments match those of other species. Anim. Cogn..

[30] Davis H. (1984). Discrimination of the number three by a raccoon (*Procyon lotor*). Anim. Learn. Behav..

[31] Abramson J.Z., Hernandez-Lloreda V., Call J., Colmenares F. (2011). Relative quantity judgements in South American sea lions (*Otaria flavescens*). Anim. Cogn..

[32] Rubenstein D.I., Hohmann M.E. (1989). Parasites and social behavior of island feral horses. Oikos.

[33] Feigenson L., Dehaene S., Spelke E.S. (2004). Core systems of number. Trends Cogn. Sci..

[34] Bisazza A., Tagliapietra C., Bertolucci C., Foà A., Agrillo C. (2014). Non-visual numerical discrimination in a blind cavefish (*Phreatichthys andruzzii*). J. Exp. Biol..

[35] Pfungst O. (1911). Clever Hans (The Horse of Mr. vonOsten): A Contribution to Experimental Animal and Human Psychology.

[36] Uller C., Lewis J. (2009). Horses (*Equus caballus*) select the greater of two quantities in small numerical contrasts. Anim. Cogn..

[37] Henselek Y., Fischer J., Schloegl C. (2012). Does the stimulus type influence horses’ performance in a quantity discrimination task?. Front. Psychol..

[38] Agrillo C., Bisazza A. (2014). Spontaneous *versus* trained numerical abilities. A comparison between the two main tools to study numerical competence in non-human animals. J. Neurosci. Meth..

[39] Agrillo C., Miletto Petrazzini M.E. (2012). The importance of replication in comparative psychology: the lesson of elephant quantity judgments. Front. Psychol..

[40] Agrillo C., Dadda M. (2007). Discrimination of the larger shoal in the poeciliid fish *Girardinus falcatus*. Ethol. Ecol. Evol..

[41] Pepperberg I.M., Brezinsky M.V. (1991). Acquisition of a relative class concept by an African gray parrot (*Psittacus erithacus*): Discriminations based on relative size. J. Comp. Psychol..

[42] Agrillo C., Piffer L. (2012). Musicians outperform nonmusicians in magnitude estimation: Evidence of a common processing mechanism for time, space and numbers. Q. J. Exp. Psychol..

[43] Agrillo C., Miletto Petrazzini M.E., Tagliapietra C., Bisazza A. (2012). Inter-specific differences in numerical abilities among teleost fish. Front. Psychol..

[44] Brannon E.M., Terrace H.S. (1998). Ordering of the numerosities 1 to 9 by monkeys. Science.

[45] Gebuis T., Reynvoet B. (2012). The role of visual information in numerosity estimation. PLoS One.

[46] Feigenson L., Carey S., Hauser M.D. (2002). The representations underlying infants’ choice of more: Object-files *versus* analog magnitudes. Psychol. Sci..

[47] Stevens J.R., Wood J.N., Hauser M.D. (2007). When quantity trumps number: Discrimination experiments in cotton-top tamarins (*Saguinus oedipus*) and common marmosets (*Callithrix jacchus*). Anim. Cogn..

[48] Agrillo C., Cohen Kadosh R., Dowker A. (2014). Numerical and arithmetic abilities in non-primate species. The Oxford Handbook of Numerical Cognition.

[49] Biro D., Matsuzawa T. (2001). Use of numerical symbols by the chimpanzee (*Pan troglodytes*): Cardinals, ordinals and the introduction of zero. Anim. Cogn..

[50] Beran M.J., Perdue B.M., Bramlett J.L., Menzel C.R., Evans T.A. (2012). Prospective memory in a language-trained chimpanzee (*Pan troglodytes*). Learn. Motiv..

[51] Patel A.D., Iversen J.R., Bregman M.R., Schulz I. (2009). Experimental evidence for synchronization to a musical beat in a nonhuman animal. Curr. Biol..

[52] Corkin S. (2002). What’s new with the amnesic patient HM?. Nat. Rev. Neurosci..

[53] Crawford J.R., Garthwaite P.H. (2012). Single-case research in neuropsychology: A comparison of five forms of t-test for comparing a case to controls. Cortex.

[54] Miklósi Á., Kubinyi E., Topál J., Gácsi M., Virányi Z., Csányi V. (2003). A simple reason for a big difference: Wolves do not look back at humans, but dogs do. Curr. Biol..

[55] Udell M.A., Dorey N.R., Wynne C.D. (2008). Wolves outperform dogs in following human social cues. Anim. Behav..

